# Changes in shift patterns due to the COVID-19 pandemic: a prospective cohort study of the intensive care nursing staff in hospitals in Stockholm

**DOI:** 10.1016/j.ijnsa.2025.100408

**Published:** 2025-08-12

**Authors:** Emma Brulin, Emelie Thunqvist, Per Gustavsson, Carolina Bigert, Tove Nilsson, Seth Addo, Abid Lashari

**Affiliations:** a Unit of Occupational Medicine, Institute of Environmental Medicine, Karolinska Institutet, Solna, Sweden; b Center for Occupational and Environmental Medicine, Region Stockholm, Stockholm, Sweden

**Keywords:** COVID-19 pandemic, ICU, nursing staff, shift work, night work, intensive care unit

## Abstract

**Background:**

It is well known that the COVID-19 pandemic placed pressure on healthcare, leading to increased job demands for intensive care unit (ICU) nursing staff and possibly also longer working hours. Potential changes in shift patterns for ICU nursing staff are important to study as numerous studies collectively indicate that certain shift patterns, especially night shifts, increase the risk of developing various diseases, such as diabetes, cardiovascular diseases, and preterm birth among nursing staff.

**Objective:**

To inform crisis planning, the aim was to investigate potential changes in shift patterns among ICU nursing staff working in public hospitals in Stockholm during the pandemic (March 1, 2020-May 31, 2022). Specifically, we investigated changes in the number of day, evening, night, and long shifts; quick returns from evening and night shifts; and mean monthly work hours corresponding to each peak of COVID-19.

**Design:**

A prospective cohort study

**Setting:**

ICU in four public hospitals in Stockholm

**Participants:**

Nursing staff, i.e., nurses, specialist nurses, and assistant nurses working in the ICU (*n* = 1208) between 1 January 2017 and 31 May 2022.

**Method:**

Data were obtained from a computerised administrative employee register in Region Stockholm, which includes detailed information on hours worked. Interrupted time series regressions, modelled with three interruptions, one for each peak of COVID-19 patient influx, were used to investigate whether COVID-19 had an impact on shift patterns.

**Results:**

Results showed that the first peak of COVID-19 led to an immediate increase in the number of night shifts by 673.15 shifts (95 % confidence interval (CI): 487.25–859.04), quick returns from night shifts by 443.44 (CI 265.03–621.85) and ≥3 consecutive night shifts by 201.51 (CI 116.57–286.46). Following the first peak, both the number of night shifts and quick returns from night shifts declined. At peak two, the number of night shifts and quick return from night shifts increased by 450.01 (CI 285.85–614.18) and 397.45 (CI 220.43–574.46), respectively. At the third peak, there was an immediate decrease in the number of working days in a row. However, from the second month, a statistically significant upward trend was observed in both the number of night shifts and quick return from night shifts.

**Conclusions:**

During the COVID-19 pandemic, (ICU nursing staff experienced an increase in both the number of night shifts and quick returns from night shifts. These types of shifts may lead to adverse health effects and should be minimised. Healthcare organisations should prepare for shift schedules during times of strain to prevent an increase in hazardous shifts.

**Study registration:**

Not registered

**Contribution of the paper**

What is already known

• The workload increased for nursing staff in general and intensive care nursing staff in particular during the COVID-19 pandemic.
• Few studies have explored potential changes in shift work due to the pandemic.
• Shift and night work can lead to adverse health outcomes.

What this paper adds

• During the pandemic, intensive care unit nursing staff in Stockholm worked more night shifts and had more quick returns from the night shifts than before.
• The first two peaks led to sharp increases in night shifts, while at the third peak, there was a decrease, followed by an upward trend.
• This increase may be hazardous, and measures are needed to reduce the increase in night shifts and quick returns from night shifts during a crisis.

Alt-text: Unlabelled box

## 1 Introduction

Extensive literature has researched frontline nursing staff, including those working in intensive care units (ICUs), highlighting the extensive workloads, and high demands during the COVID-19 pandemic (Morgantini et al., 2020; Harris et al., 2021; Akerstrom et al., 2022; Nikbakht Nasrabadi et al., 2022; Nilsson et al., 2022). As the virus spread rapidly, healthcare organisations were compelled to promptly adapt work arrangements for healthcare workers, including transferring to other care units, higher staff coverage, and time-consuming protection procedures (Akerstrom et al., 2022; Nilsson et al., 2022; Doleman et al., 2023). Under these circumstances, it is highly likely that the increased burden on healthcare services, in addition to rising work demands, also led to changes in working time scheduling and an increase in working hours. (Morgantini et al., 2020). Given the strong evidence that shift and nightwork adversely affect health and safety in both the short and long term, it is crucial to determine whether the nursing staff were unduly exposed to hazardous shift work.

While shift work is necessary for 24/7 healthcare, shift and night work interfere with several areas of life, such as the disturbance of circadian rhythms, workability, social relationships, and health (Costa, 2015). Numerous studies, including meta-analyses, collectively indicate that shift work involving night shifts increases the risk of various diseases, such as type 2 diabetes (Kivimäki, Virtanen, et al., 2015; Viklund et al., 2023) and cardiovascular disease (Kivimäki, Jokela, et al., 2015; Li et al., 2016; Bigert et al., 2022; Kader, Selander, et al., 2022), as well as preterm birth for pregnant nursing staff (Kader, Bigert, et al., 2022). There are also strong indications that night work increases the risk of various forms of cancer, especially breast cancer (Dun et al., 2020; Manouchehri et al., 2021; Gustavsson et al., 2023). Moreover, there is also evidence that quick returns from night shifts (<28 h) increase the risk of stroke and from evening shifts (<11 h between shifts) reduce sleep duration and quality (Karhula et al., 2013; Costa, 2015; Vedaa et al., 2016; Härmä et al., 2018; Härmä, Kecklund and Tucker, 2024), and increase the risk of accidents and sickness absence (Vedaa et al., 2019). Systematic reviews show that long shifts (>12 h) and weeks are related to cardiovascular and metabolic diseases (Kivimäki, Jokela, et al., 2015).

Shift and night work have also been associated with common mental disorders. Both quick returns from evening shifts as well as night shifts are associated with sleep disorders, burnout, and mental health problems (Virtanen et al., 2011; Ferri et al., 2016; Cheng and Cheng, 2017). Night workers in healthcare who already suffered from common mental disorders were twice as likely to recover from their illness if they switched to daytime work compared with those who continued to work night shifts (Beltagy et al., 2018). Switching from shift work to day work is also associated with decreased fatigue (Härmä et al., 2019). Working long hours is also a risk factor for the development of stress and mental health problems (Virtanen et al., 2011; Ge et al., 2023).

Furthermore, shift and night work often challenge the balance between work and private life (Dahlgren et al., 2016; Karhula et al., 2017). During the first wave of the COVID-19 pandemic, the imbalance between work and private life increased for health and social care workers compared to other sectors (Brulin, Leineweber and Peristera, 2022). This imbalance is, in turn, a known risk factor for burnout (Gynning et al., 2024) and sickness absence (Hagqvist, Lidwall and Leineweber, 2022).

Although research points to a range of negative impacts from different shift types, few studies have explored potential changes in shift types before and during the COVID-19 pandemic and whether potential high-risk shifts increased. A cross-sectional survey of nurses in Norway shows that a fourth of the included nurses reported changes in their schedule due to the pandemic, and that most shift changes were to long shifts (>12 h) (Djupedal et al., 2022). A study from Slovenia used nurses' schedules comparing shift work in 2019 to 2021 in a sample of 24 nurses (Peršolja, 2022). The study showed a statistically significant increase in working time in 2021 compared to 2019, with increased evening and night shifts.

A limitation of the few previous studies is the use of self-reported information on working hours and schedules. Using detailed working time data for each hour worked, this study aims to inform stakeholders and policymakers by exploring whether and how shift schedules changed compared to before the COVID-19 pandemic for intensive care unit (ICU) nursing staff working in public hospitals in the Stockholm area. Specifically, we investigated changes in the number of day, evening, night, and long shifts, quick returns from evening and night shifts, mean monthly work hours, and the number of working days in a row. Furthermore, we analysed potential fluctuations in shift patterns throughout the peaks of COVID-19.

## 2 Method

Data for this study derive from a computerised administrative employee register (HEROMA) in Region Stockholm between 1 January 2017 and 31 May 2022. HEROMA data includes detailed individual information on working hours day by day, including the exact start and end times for each individual shift. The HEROMA register includes both in- and outpatient care services, such as emergency hospitals, local medical centres, family doctors, and maternity clinics. We restricted the study sample to public hospital (*n* = 4) nursing staff, i.e., nurses, specialist nurses, and assistant nurses working in the ICU at any point during the study period. All hospitals were similarly impacted by the COVID-19 pandemic.

### 2.1 Assessment of shift work measurements

The method for aggregation and classification of shift schedule patterns based on register-based working hours for healthcare employees was previously developed in Finland (Härmä et al., 2015) and utilised specifically for HEROMA in our studies investigating shift and night work and the risk of preterm birth (Kader, Bigert, et al., 2022), cerebrovascular disease (Bigert et al., 2022), ischemic heart disease and atrial fibrillation (Kader, Selander, et al., 2022) and type 2 diabetes and hypertension (Viklund et al., 2023). According to this, all shifts were classified as follows (Table 1): day shifts (starts after 06:00 and ends no later than 18:00 and lasts for at least 4 h); evening shifts (starts after 12:00 and ends later than 18:00, but not later than 22:00 and lasts for at least 4 h); and night shifts (≥3 h of work within 22:00–06:00).

**Table 1:** Shift types and working times.

Shift type	
Day shift	Starts after 06:00 and ends no later than 18:00, and lasts for at least 4 h.
Evening shift	Starts after 12:00 and ends later than 18:00, but not later than 22:00 and lasts for at least 4 h
Night shift	≥3 h of work within 22:00–06:00
Long shift	>12 h
Quick return from evening shift	<11 h to the next shift
Quick return from night shift	<28 h to the next shift

Furthermore, long shifts were defined as shifts longer than 12 h. Quick returns from night shifts were defined as <28 h between the end of a night shift and the beginning of the following shift (the following shift could be a day, evening, or night shift), and quick returns from evening shifts were defined as <11 h between the end of an evening shift and the beginning of the following shift (day or evening shift). Mean monthly working hours were defined as the mean of all worked hours every month. Thereto, we measured the number of consecutive working days in a row.

The number of day, evening, night, and long shifts, quick returns from evening and night shifts, ≥3 consecutive nights, the number of working days in a row, and mean monthly working hours were calculated monthly.

### 2.2 COVID-19 cases

The Swedish National Board of Health and Welfare collected data on the total number of COVID-19 patients in hospitals (Socialstyrelsen, 2024). The data includes the number of unique patients with COVID-19 who were hospitalised, including ICU, in Region Stockholm from April 2020 (no earlier data available) to May 2022.

### 2.3 Analytical strategy

In order to show the trend, its linearity, and stability of the results over time, we primarily aggregated the data by month. We selected one-month intervals because they provided enough information for each interval and enough intervals within each period to enable modelling and visualisation of the relationship over time. Plots show the number of each shift type for each month between January 2017 and May 2022.

**Fig. 1:**
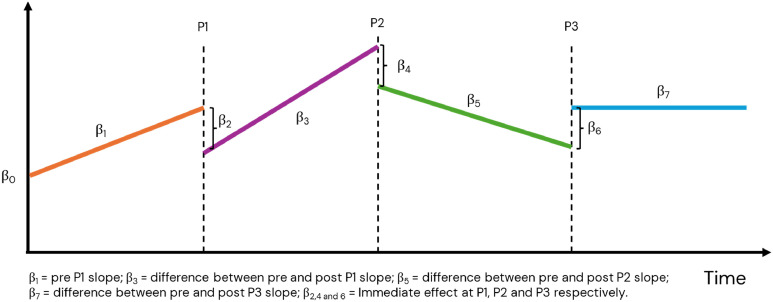
An illustration of the function of the interrupted time series with three interruptions and the corresponding β values. The lines and β values are hypothetical to show how the method is used in this study.

Segmented regression of interrupted time series was conducted to determine whether the COVID-19 pandemic impacted the numbers and trends of the different types of shifts. The interrupted time series was modelled with three interruptions (Fig. 1), one for each peak (P), i.e., the start of the increase in COVID-19 patient influx (P1: March 1st 2020; P2: October 1st 2020; P3: November 1st 2021). We used Newey-West adjustment of the standard errors to account for autocorrelation. An interrupted time series is useful for evaluating a wide range of public health interventions or the effect of unplanned events at a population level (Lopez Bernal, Cummins and Gasparrini, 2016), such as COVID-19. The standard regression of the interrupted time series model is specified as follows:

$$\mathrm{Yt}=\beta_{0}+\beta_{1}T_{t}+\beta_{2}X_{t}+\beta_{3}P_{t}+\beta_{4}X_{1t}+\beta_{5}P_{1t}+\beta_{6}X_{2t}+\beta_{7}P_{2t}+\varepsilon_{t}$$

Yt is the outcome representing the change in the monthly numbers of shifts at time t; T refers to the time elapsed since the beginning of the study in months; X_t_, X_1t,_ X_2t_ are virtual (indicator) variables representing the three COVID-19 peaks (the period before each COVID-19 peak is 0; otherwise, it is 1); P_t_, P_1__t_, and P_2t_ denotes the number of months passed after the start of the first, second and third peak, respectively; β_0_ is the baseline level. The error term ε_t_ allows for deviation from the fitted model. Fig. 1 illustrates the formula of the segmented regression interrupted time series. In this study, the β indicates the number of changed shifts.

### 2.4 Ethics

The Ethical Review Board in Stockholm (2016/2490–31; 2017/1157–32) and the National Ethical Review Authority granted ethical permission for the study (2022–06828–02). Informed consent for participation in the study is not requested for register-based research. However, information about opting out was posted on the internal website, along with contact information for the researchers.

## 3 Results

**Fig. 2:**
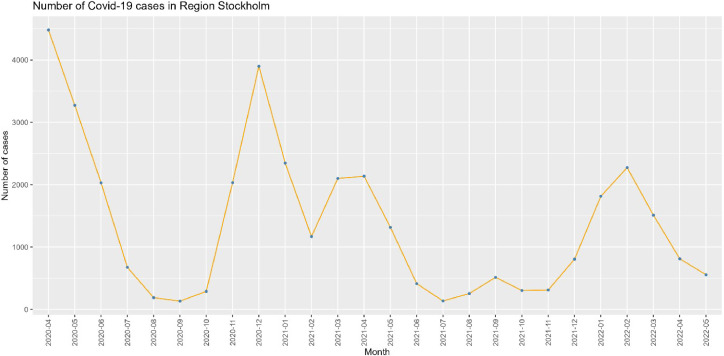
The number of unique hospitalised COVID-19 patients in Stockholm is based on data from the National Board of Social Affairs and Health (Data only available from April 2020 to May 2022).

The study sample consisted of 1208 employees, comprising 1000 (82.8 %) women, 208 (17.2 %) men, 633 (52.4 %) nurses, and 575 (47.6 %) assistant nurses. First, in Fig. 2, we share an overview of the number of hospitalised COVID-19 patients in Stockholm. There were three main peaks: March to May 2020 (P1), November 2020 to May 2021 (with a short drop in February; P2) and December 2021 to April 2022 (P3). The interruptions were set at the start of each peak. At large, there is a reduction in the number of COVID cases with each peak.

**Fig. 3:**
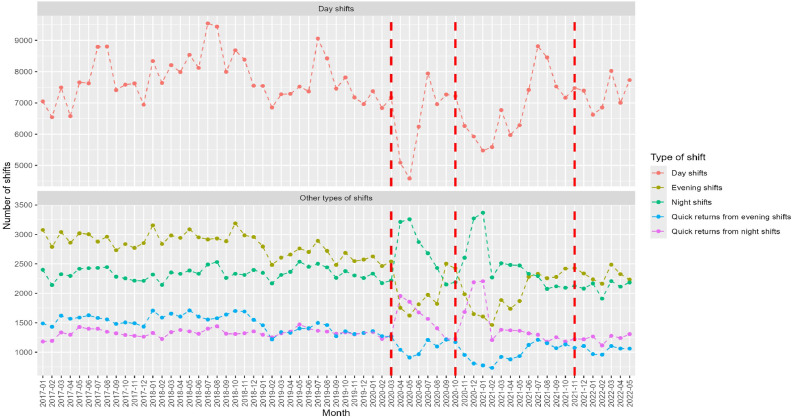
Number of day, evening and night shifts, and quick returns from night and evening shifts among ICU nursing staff in Stockholm public hospitals from January 2017 to May 2022. Due to the high number of day shifts, they are presented separately to enhance readability. The vertical red lines indicate the start of each COVID-19 peak, i.e., the measured interruption.

**Fig. 4:**
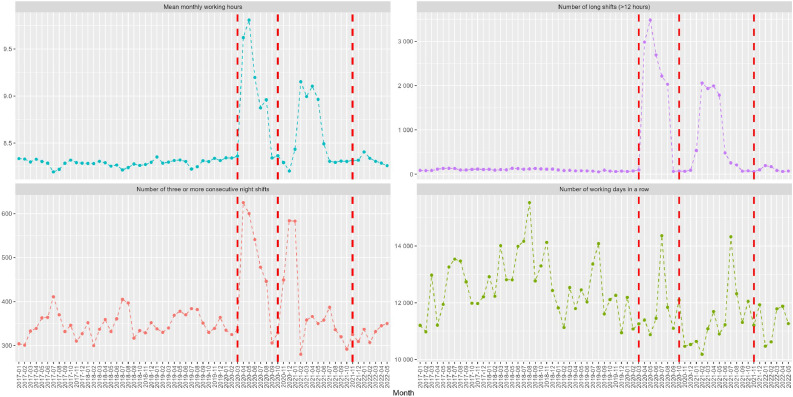
Number of long shifts, days in a row, ≥3consecutive night shifts and mean monthly working hours among ICU nursing staff in Stockholm public hospitals from January 2017 to May 2022. The vertical red lines indicate the start of each COVID-19 peak, i.e., the measured interruption.

In Fig. 3, Fig. 4, we plotted the monthly number of the studied shift patterns. Across all shift types, we observe periodic changes in the shift schedules for the summer months each year, specifically July and August, which are comparable to summer holidays. Corresponding to the first two peaks of COVID, there was a decrease in the number of day and evening shifts, while the number of night shifts, ≥3 consecutive nights, quick returns from night shifts, and monthly working hours increased. Before COVID-19, there were a few long shifts that increased statistically significantly in the first peak. Overall, the most extensive changes were observed during the first peak.

The interrupted time series results in Table 2 showed that at P1, there was a statistically significant and immediate decrease in the number of day shifts (β = −2352.44, CI=−3891.57, −813.32), evening shifts (β = −680.89, CI = −1358.93, −2.86), quick returns from evening shifts (β = −303.51 CI= −587.99, −19.03), and number of working days in a row (β = −1258.63, CI = −2288.75, −228.50). There was a statistically significant increase in the number of night shifts (β = 673.15, CI = 487.25, 859.04), quick returns from night shifts (β = 443.44, CI = 265.03, 621.85), and ≥3 consecutive nights (β=201.51; CI =116.57, 286.46). After the first month, the number of day shifts increased (β = 264.67; CI = 32.53, 496.81) while the number of night shifts, ≥3 consecutive nights, and quick return from night shifts per month decreased statistically significantly and gradually over time, while no statistically significant changes were observed for the other shift types.

**Table 2:** The statistical results of interrupted time series regression analyses of the impact of the COVID-19 pandemic on the number of shifts in the ICU by shift type subgroups.

		Peak 1		Peak 2		Peak 3	
	Pre-pandemic period	Rate Change^a^	COVID-19 trend^b^	Rate Change^a^	COVID-19 trend^b^	Rate Change^a^	COVID-19 trend^b^
Day shift β	−0.78	**−2352.44**	**264.67**	**−1547.80**	−102.77	−702.74	−111.05
95 % CI	−31.80, 20.56	−**3891.57, −813.32**	**32.53, 496.81**	**−2495.47, −600.12**	−390.39, 184.84	−1436.45, 30.98	−262.52, 40.42
Evening shift β	**−10.86**	**−680.89**	24.25	−326.38	28.88	98.63	−54.81
95 % CI	**−15.03, −6.69**	**−1358.93, −2.86**	−112.16, 160.66	−833.53, 180.78	−139.01, 196.77	−166.94, 364.19	−119.20, 9.58
Night shift β	0.61	**673.15**	**−84.29**	**450.01**	23.72	−35.41	**70.03**
95 % CI	−0.61, 1.83	**487.25, 859.04**	**−124.73, −43.85**	**285.85, 614.18**	−18.80, 66.24	−100.69, 29.88	**56.21, 83.84**
Long shift (>12 h) β	**−1.16**	2327.21	−115.34	−707.66	96.55	−493.98	14.73
95 % CI	**−1.98, −0.33**	−129.94, 4784.36	−626.49, 395.80	−2428.74, 1013.41	−464.71, 657.82	−1419.14, 431.19	−133.73, 163.19
QR from night shifts β	0.85	**443.44**	**−56.35**	**397.45**	6.08	39.2	**60.24**
95 % CI	−0.50, 2.20	**265.03, 621.85**	**−95.78, −16.92**	**220.43, 574.46**	−35.67, 47.83	−21.76, 100.16	**45.15, 75.33**
QR from evenings β	**−7.09**	**−303.51**	16.16	**−285.15**	11.9	−69.64	−20.43
95 % CI	**−10.46, −3.72**	**−587.99, −19.03**	−31.27, 63.59	**−534.79, −35.51**	−46.54, 70.33	−223.48, 84.20	−51.00, 10.13
≥3 consecutive nights β	0.42	**201.51**	**−20.67**	57.32	7.62	0.79	**17.70**
95 % CI	−0.04, 0.88	**116.57, 286.46**	**−39.79, −1.55**	−8.93, 123.56	−11.79, 27.03	−14.66, 16.25	**13.84, 21.57**
Number of working days in a row β	−6.76	**−1258.63**	146.65	**−1651.97**	−7.69	**−1129.78**	−82.6
95 % CI	−48.60, 35.09	**−2288.75, −228.50**	−83.87, 377.16	**−2952.77, −351.17**	−224.50, 209.13	**−1859.11, −400.45**	−214.19, 49.00
Mean monthly working hours β	−86.01	2639.06	380.90	−12,337.39	226.42	−9116.7	−189.42
95 % CI	−370.61, 198.59	−9733.59, 15,011.71	−2775.01, 3536.80	−24,897.25, 222.46	−3111.28, 3564.12	−20,027.48, 1794.09	−1222.53, 843.69

Bold indicates statistically significant β values with *p* < 0.05; QR= quick return.

a Peak interruption.

b Differences between slopes before and after interruption.

At the second peak, there was again a statistically significant increase in the number of night shifts (β = 450.01, CI = 285.85, 614.18) and quick returns from night shifts (β = 397.45, CI = 220.43; 574.46), and a decrease in quick returns from evening shifts (β = −285.15, CI = –534.79, −35.51) as well as in number of working days in a row (β = −1651.97, CI = −2952.77, −351.17). There were no statistically significant slope changes in the months that followed the second peak, indicating that night shifts and quick returns from night shifts remained elevated.

Immediately at the third peak, there was a statistically significant decrease in the number of working days in a row (β = −1129.78, CI = −1859.11, −400.45), but no statistically significant changes were observed for other shift types. However, a statistically significant upward trend was observed after the first month in the number of night shifts (β = 70.03, CI = 56.21, 83.84), as well as quick returns from night shifts (β = 60.24, CI = 45.15, 75.33) and ≥3 consecutive nights (β = 15.49, CI = 3.25, 27.73).

## 4 Discussion

This study utilised a large population cohort, including detailed information on working time, to investigate how shift patterns changed for ICU nursing staff in public hospitals in Region Stockholm, in response to the COVID-19 pandemic. Specifically, we investigated changes in the number of day, evening, night, and long shifts, quick returns from evening and night shifts, working days in a row, and mean monthly working hours. During the COVID-19 pandemic, ICU nursing staff in public hospitals in Stockholm worked more night shifts than before, with sharp increases at the first two peaks and a slow upward trend at the last peak, also leading to more quick returns from night shifts. At the first peak, the number of day and evening shifts, quick returns from evening shifts, and consecutive days worked decreased. Our results contribute to those from Slovenia utilising schedule data for two years and Norway using a cross-sectional survey that indicated an increase in working time, especially in long, evening and night shifts, at the start of the pandemic (Djupedal et al., 2022; Peršolja, 2022) and that there was an initial increase in working time, which decreased and stabilised over time (Shen et al., 2022).

Considering that night shifts, as well as quick returns from night shifts, are well-recognized risk factors for diseases, including cardiovascular disease, cancer, type 2 diabetes (Kivimäki, Jokela, et al., 2015; Vedaa et al., 2016; Dun et al., 2020; Manouchehri et al., 2021; Kader, Selander, et al., 2022; Gustavsson et al., 2023; Viklund et al., 2023; Härmä, Kecklund and Tucker, 2024), stress and mental health problems (Virtanen et al., 2011; Ge et al., 2023), and sleep disorders (Karhula et al., 2013; Costa, 2015; Vedaa et al., 2016; Härmä et al., 2018; Härmä, Kecklund and Tucker, 2024; Nilsson et al., 2025), the health of ICU nursing staff is a matter of concern. Furthermore, a longitudinal cohort study by Härmä et al. (2019) showed that changing from day work to any shift work was associated with an increased risk for long sleeps, indicating an increased need for recovery after six years when compared with those staying in day work. This suggests that for nurses who transitioned from working days to working nights during the pandemic, there may be adverse health outcomes.

In Sweden, night work is, in principle, prohibited and permissible under the Working Hours Act (1982:673). Work during the night is only allowed if it is “necessary due to the nature of the work, the needs of the public or other special circumstances”, such as in the healthcare sector. The EU directives (2003/88/EC) stipulate that night workers are entitled to a free health assessment before being assigned to night work and at regular intervals thereafter. In Sweden, these directives are implemented, specifying that night workers should undergo or be offered a medical assessment when they begin working nights and then periodically (Arbetsmiljöverket, 2025). It was unlikely that these medical assessments were adjusted to the increase in or introduction of night shifts during COVID-19. To limit adverse health effects from night work in future crises with high impact on healthcare, scheduling guidelines and recommendations on medical assessment should be included in preparedness plans.

The results should be regarded against the backdrop that the general workload was high during the pandemic (Morgantini et al., 2020; Foli et al., 2021; Bucca et al., 2022). A comparative analysis of nurses' burnout before and during the COVID-19 pandemic by Ge et al. (2023) showed that nurses' burnout rates increased during COVID-19. The study by Ge et al. (2023) discusses that even before the outbreak of COVID-19, nurses reported high burnout due to, for example, shifts and overnight work. Further, a Norwegian study found that changes in the work schedule due to the pandemic increased the risk of turnover intention (Djupedal et al., 2022). When working evening or night shifts, there is a risk of an accumulation of hazardous psychosocial exposures, especially in terms of low job control, low social support from leaders, exposure to physical and psychological violence, and physical workload (Nabe-Nielsen et al., 2009; Lindahl Norberg and Falkstedt, 2023). Thus, the combination of high workload and night work may cause vicious circles leading to turnover or sick leave. Also, since the end of the pandemic, healthcare demands have remained high, with significant hospitalisation rates from seasonal flu and other viruses, a care depth, i.e., the elective care that was not carried out due to the pandemic, and increasing rates of healthcare staff with poor mental health and sick leave (Försäkringskassan, 2022; Brulin et al., 2023).

Although increasing working hours during a crisis might be necessary, the health of the nursing staff must be prioritised. The adverse health effects of working long hours and night work with a high workload might, in turn, lead to turnover or sickness absence (Ropponen et al., 2019; Larsen et al., 2020). These problems thus result in great personal suffering for those affected and may also negatively impact patient safety and healthcare provision (Virtanen et al., 2009; Montgomery et al., 2011, 2020; Teoh, Hassard and Cox, 2021). The clinical implications of this study are that healthcare organisations must provide strategies for shift scheduling, recovery, and ease of workload in pandemic preparedness plans to mitigate any potential adverse health effects from working in 24/7 healthcare. These plans should also include recommendations on medical assessments. The results may also be transferred to the general preparedness for any crisis with a great impact on healthcare.

### 4.1 Strengths and limitations

This study's primary strength is its reliance on objectively measured working time from employee registers, which has not been used before to evaluate changes in shift patterns in the healthcare sector (Djupedal et al., 2022; Peršolja, 2022; Shen et al., 2022). The data is very detailed, with individual day-to-day information on different types of shift work and working hours. Since no data needed to be obtained from the employees themselves, this eliminates the risk that people will forget details about their working hours.

Another advantage of our study is that the interrupted time series calculated the growth rate of shift rate before and after COVID-19, which more intuitively showed the influence of the pandemic on shift rates.

A limitation of our study is that we did not measure any health outcomes, which makes it hard to conclude which effects the changes in shift rate have had on the healthcare personnel, but previous research has seen that shift and night work are well-recognised risk factors for health and well-being. Since this is a prospective cohort study of the changes in shift and night work schedules during the COVID-19 pandemic for nursing staff in hospitals in Region Stockholm, future research, which include measures of outcomes, is needed.

## 5 Concluding remarks

This study shows that the number of night shifts and quick returns from night shifts increased during the COVID-19 pandemic for ICU nursing staff in Stockholm. Overall, the most extensive changes in shift patterns were observed during the first peak of COVID-19. Given the increasing risk of adverse health effects associated with working night shifts and quick returns from night shifts, there is a pressing need for national policies and guidelines regarding shift work during crises that significantly impact healthcare to prevent excessive night work. Also, healthcare organisations should monitor shift patterns during crises to limit the potential increase in hazardous shifts. Furthermore, preparedness plans should include recommendations on how to schedule healthy shifts during crises and provide recommendations for medical assessments.

**Data availability**: Data sharing is prohibited due to the research's ethical regulations. Aggregated data may be shared upon request by the authors.

## Funding sources

This study was conducted without funding.

## Declaration of generative AI and AI-assisted technologies in the writing process

During the preparation of this work, the authors used Grammarly to improve the readability and language of the manuscript. After using this tool/service, the authors reviewed and edited the content as needed and took full responsibility for the content of the published article.

## CRediT authorship contribution statement

**Emma Brulin:** Writing – review & editing, Writing – original draft, Validation, Supervision, Resources, Project administration, Conceptualization. **Emelie Thunqvist:** Writing – review & editing, Writing – original draft, Conceptualization. **Per Gustavsson:** Writing – review & editing. **Carolina Bigert:** Writing – review & editing. **Tove Nilsson:** Writing – review & editing. **Seth Addo:** Writing – review & editing. **Abid Lashari:** Writing – review & editing, Validation, Formal analysis, Data curation.

## Declaration of competing interest

Authors have nothing to declare.
